# A prediction model for xerostomia in locoregionally advanced nasopharyngeal carcinoma patients receiving radical radiotherapy

**DOI:** 10.1186/s12903-022-02269-0

**Published:** 2022-06-17

**Authors:** Minying Li, Jingjing Zhang, Yawen Zha, Yani Li, Bingshuang Hu, Siming Zheng, Jiaxiong Zhou

**Affiliations:** Department of Oncology Radiotherapy, Zhongshan City People’s Hospital, No.2 Sunwen Middle Road, Shiqi District, Zhongshan City, 528403 Guangdong China

**Keywords:** Xerostomia, Nasopharyngeal carcinoma, Prediction

## Abstract

**Background:**

This study was to evaluate the predictors of xerostomia and Grade 3 xerostomia in locoregionally advanced nasopharyngeal carcinoma (NPC) patients receiving radical radiotherapy and establish prediction models for xerostomia and Grade 3 xerostomia based on the predictors.

**Methods:**

Totally, 365 patients with locoregionally advanced NPC who underwent radical radiotherapy were randomly divided into the training set (n = 255) and the testing set (n = 110) at a ratio of 7:3. All variables were included in the least absolute shrinkage and selection operator regression to screen out the potential predictors for xerostomia as well as the Grade 3 xerostomia in locoregionally advanced NPC patients receiving radical radiotherapy. The random forest (RF), a decision tree classifier (DTC), and extreme-gradient boosting (XGB) models were constructed. The sensitivity, specificity, positive predictive value (PPV), negative predictive value (NPV), area under the curve (AUC) and accuracy were analyzed to evaluate the predictive performance of the models.

**Results:**

In the RF model for predicting xerostomia, the sensitivity was 1.000 (95%CI 1.000–1.000), the PPV was 0.990 (95%CI 0.975–1.000), the NPV was 1.000 (95%CI 1.000–1.000), the AUC was 0.999 (95%CI 0.997–1.000) and the accuracy was 0.992 (95%CI 0.981–1.000) in the training set. The sensitivity was 0.933 (95%CI 0.880–0.985), the PPV was 0.933 (95%CI 0.880–0.985), and the AUC was 0.915 (95%CI 0.860–0.970) in the testing set. Hypertension, age, total radiotherapy dose, dose at 50% of the left parotid volume, mean dose to right parotid gland, mean dose to oral cavity, and course of induction chemotherapy were important variables associated with the risk of xerostomia in locoregionally advanced NPC patients receiving radical radiotherapy. The AUC of DTC model for predicting xerostomia was 0.769 (95%CI 0.666–0.872) in the testing set. The AUC of the XGB model for predicting xerostomia was 0.834 (0.753–0.916) in the testing set. The RF model showed the good predictive ability with the AUC of 0.986 (95%CI 0.972–1.000) in the training set, and 0.766 (95%CI 0.626–0.905) in the testing set for identifying patients who at high risk of Grade 3 xerostomia in those with high risk of xerostomia.

**Conclusions:**

An RF model for predicting xerostomia in locoregionally advanced NPC patients receiving radical radiotherapy and an RF model for predicting Grade 3 xerostomia in those with high risk of xerostomia showed good predictive ability.

## Background

Nasopharyngeal carcinoma (NPC) is a prevalent malignant tumor in endemic regions, with the highest incidence rate among malignant tumors of the ear, nose and throat [1]. It has been reported about 130,000 patients worldwide in 2018 and 1/2 of the cases were from China [2]. In China, NPC is common in Southeast China, with 15–50 cases in 100,000 people annually [3, 4]. NPC is manifested as blood in the nose, hearing loss, nasal congestion, headache [5]. Among 87,000 new cases annually, more than 70% of them are staged at locoregionally advanced NPC [6]. During the past 20 years, the combination of radiotherapy techniques, such as Intensity Modulated Radiation Therapy (IMRT) has greatly improved the survival rate of NPC patients with local tumor control rates reaching more than 90% [7]. Radiotherapy can also cause radiation damage to normal tissues in the radiation part, resulting in different degrees of short-term and long-term radiation complications [8]. NPC patients receiving radiation therapy may suffer from complications including radio-pulmonary lesion, radiation esophagitis, radiodermatitis, xerostomia, radioactive parotitis and so on [9, 10].

Xerostomia is one of the most common complications of radiotherapy in NPC patients [11]. The incidence of xerostomia was reported to be over 30% after IMRT treatment [12]. In NPC patients receiving the conventional external irradiation treatment, the function of parotid gland is seriously damaged and the salivary secretion is decreased, due to the high dose irradiation to parotid gland, which lead to xerostomia [13]. Radiotherapy can also damage the cellular enzyme system and cause a severe inflammatory response in the parotid gland, which is also the main cause of xerostomia [14]. Although xerostomia patients slowly recovered their saliva secretion post treatment, xerostomia remains consistent over time in about 40% of patients [15]. The long-term xerostomia can adversely affect teeth, language, swallowing, and chewing [16]. Most NPC patients with xerostomia have difficulties in eating normally and experience discomfort and pain when chewing and swallowing food [17]. Some patients need to drink water or soup frequently when eating, otherwise food particles will get stuck in the mouth or throat [18]. Xerostomia also decreases the overall quality of life of NPC patients by disrupting their speech and communication ability [19]. High grade of xerostomia was also reported to aggravate fatigue, sleeping domains and emotional functioning on quality of life scales [15, 20].

Since xerostomia has a significant impact on the quality of life in NPC patients, identifying predictors of xerostomia especially patients with severe xerostomia is essential to improve the prevention and treatment of it. In this study, we analyzed the factors that can predict xerostomia as well as Grade 3 xerostomia in patients with locoregionally advanced NPC receiving radical radiotherapy and established prediction models based on predictors. Patients at high risk of Grade 3 xerostomia was further predicted in those with predicted risk of xerostomia. The findings of this study might provide a guidance for clinical identification of patients who would develop xerostomia or Grade 3 xerostomia as early as possible and give appropriate interventions.

## Methods

### Study population

**Fig. 1:**
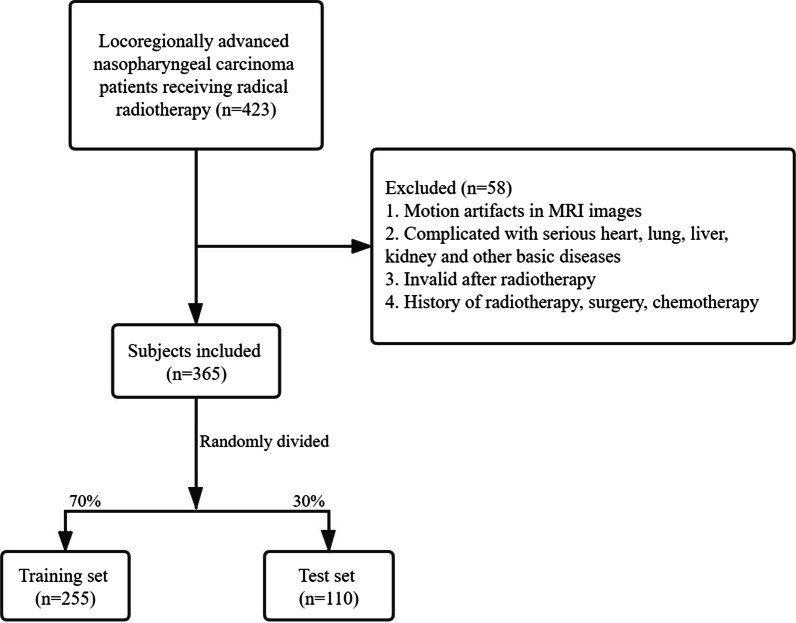
The screen process of participates in this study

This retrospective case–control study collected the data of 423 patients with locoregionally advanced NPC who underwent radical radiotherapy from Zhongshan City People’s Hospital were enrolled in our study. All patients completed the planned radiotherapy. After excluding those with motion artifacts in MRI images, complicated with serious heart, lung, liver, kidney and other basic diseases, invalid after radiotherapy and a history of radiotherapy, surgery, chemotherapy, 365 participants were finally included. Patients were randomly divided into the training set (n = 255) and the testing set (n = 110) at a ratio of 7:3 to test the model fitting effect. The screen process was shown in Fig. 1. The informed consents were obtained from the participants and this study was approved by Zhongshan City People’s Hospital (No. 2021-046).

### The diagnosis of xerostomia

The diagnosis of xerostomia was conducted according to the toxicity criteria of the Radiation Therapy Oncology Group (RTOG) and the European Organization for Research and Treatment of Cancer (EORTC) [21]. Grade 0 (normal): no obvious change compared with pre-radiotherapy; Grade 1 (slight dryness of mouth): patients with subjective dryness of mouth and soup is not necessary in eating dry food; Grade 2 (moderate dryness of mouth): soup is necessary when eating, otherwise dry food is impossible to eat; Grade 3 (complete dryness of mouth): need to wake up at night to drink water or a little long speaking will cause dryness of mouth and feel discomfort.

### Data collection

The clinical data of patients were collected from all participants including age at the time of receiving radiation treatment (years), gender, history of drinking, history of smoking, history of surgery, history of hypertension or diabetes, T stage (T1, T2, T3 and T4), N Stage (N0, N1, N2 and N3), pathological type (a differentiated non-keratinic carcinoma or an undifferentiated nonkeratinic carcinoma), radiotherapy fraction (≤ 30 fractions or > 30 fractions), dose at 50% of the left parotid volume (Gy), dose at 50% of the right parotid volume (Gy), mean dose to left parotid gland (Gy), mean dose to right parotid gland (Gy), mean dose to oral cavity (Gy), total radiotherapy dose (Gy), mode of radiotherapy [Nedaplatin (NDP), cisplatin (DDP), or others], concomitant chemoradiotherapy or not, induction chemotherapy or not, the regimens of induction chemotherapy [docetaxel + cisplatin + 5-fluorouracil (DPF), docetaxel + cisplatin + 5-fluorouracil (TPF), cisplatin + docetaxel (DP), cisplatin (TP), gemcitabine + cisplatin (GP) or others], course of induction chemotherapy, progression-free survival (PFS; month) and outcome variables (xerostomia Grade 0, Grade 1, Grade 2, and Grade 3).

### Definition of variables

Hypertension was defined considered as systolic blood pressure ≥ 140 mmHg and/or diastolic blood pressure ≥ 90 mmHg and/or as present in the subjects who had medication for hypertension at the time [22]. Diabetes was defined as subjects who had current history of diabetes mellitus and/or fasting plasma glucose concentration of 7.0 mmol/L (126 mg/dL) or higher, or 2-h post-glucose load venous plasma glucose of 11.1 mmol/L (200 mg/dL) or higher, confirmed on two occasions [23]. History of drinking was defined as drinking at least once each week for more than 3 months [24]. Dose at 50% of the left parotid volume refers to the radiotherapy dose to the 50% volume of the left parotid.

### Radiotherapy technique

All patients were in the supine position, with the connecting line of the third cervical vertebrae and the mandibular angled perpendicular to the bed and both hands naturally placed on the sides of the body. A neck and shoulder thermoplastic mask was applied for fixing the head and upper neck. CT simulation scan was performed in patients from the head to the lower edge of the clavicle and a layer thickness of 3 mm. The CT images were then imported in the Monaco® (Elekta Medical Systems, Sweden) physician workstation, on which the target area and the area of organs at risk (OARs) were delineated. The gross tumor volumes (GTVs) were divided into nasopharyngeal primary gross tumor volume (GTVnx) and neck metastatic lymph node gross tumor volume (GTVnd). The clinical target volumes (CTVs) were divided into the high-risk area (CTV1) and the low-risk area (CTV2) on the basis of tumor invasion. The various planning target volumes (PTVs) were defined from the respective target volumes extending 3 mm margins with 3D expansion, corresponding to PGTVnx, PGTVnd, PTV1 and PTV2. The OARs included the brain stem, spinal cord, temporal lobes, pituitary, optic chiasm, optic nerves, lenses, inner ears, temporomandibular joints, parotid glands, and mandible. All patients were subjected to volumetric-modulated arc therapy (VMAT). Dose optimization and calculation were analyzed via the Monaco treatment planning system. VMAT were generated by a 6 MV X-ray system, and a single or double arc design was applied according to the tumor volume and the degree of invasion. The prescribed doses were as follows: 68–72 Gy to the PGTVnx, 64–68 Gy to the PGTVnd, 60 Gy to the PTV1, and 54–56 Gy to the PTV2, in 30–33 fractions. Radiation was delivered once per day, at 5 fractions per week.

### Chemotherapy

Some patients received chemotherapy in our study, including concomitant chemotherapy with or without inductive chemotherapy. Inductive chemotherapy consisted of docetaxel + cisplatin + 5-fluorouracil (DPF), docetaxel + cisplatin + 5-fluorouracil (TPF), cisplatin + docetaxel (DP), cisplatin (TP), gemcitabine + cisplatin (GP) or others every 3 weeks for two to three cycles. Concomitant chemotherapy was cisplatin weekly (30–40 mg/m^2^) or on weeks 1, 4 and 7 (80–100 mg/m^2^) of radiotherapy.

### Statistical analysis

The measurement data of normal distribution were described as Mean ± standard deviation (Mean ± SD), and t test was applied for comparisons between groups. Non-normal data were expressed via [M (Q_1_, Q_3_)], and comparisons between groups was subjected to Mann–Whitney U rank sum test. The enumeration data were displayed as [N (%)]. Chi-square test or Fisher’s exact probability method was employed to compare differences between groups. Variables were included in the least absolute shrinkage and selection operator (LASSO) regression, with α = 0.025 as a hyperparametric screening variable, and the final remaining variables were hypertension, age, total radiotherapy dose, dose at 50% of the left parotid volume, mean dose to right parotid gland, mean dose to oral cavity, and course of induction chemotherapy. These variables were then included in a random forest (RF) model, a decision tree classifier (DTC) model, and extreme-gradient boosting (XGB) model. Furthermore, LASSO regression was applied for screen the predictors for the occurrence of Grade 3 xerostomia in the patients who had high predicted risk of xerostomia and the prediction models were also established. The predictive abilities of the models were verified using sensitivity, specificity, positive predictive value (PPV), negative predictive value (NPV), the area under the curve (AUC), and accuracy. Finally, the receiver operator characteristic curve (ROC) curve and feature importance diagram of the final model (random forest model) were drawn. Statistical tests were conducted by bilateral tests, and *P* < 0.05 was considered as statistical difference. The analysis of differences between different groups was performed using SAS v 9.4, and the statistical analysis was conducted using Python v 3.6.3.

## Results

### The baseline data of characteristics of all participants

In the present study, 365 locoregionally advanced NPC patients undergoing radical radiotherapy were involved in. The mean age of patients receiving radiation treatment was 47.69 ± 11.01 years. Among all participants, 266 (72.88%) were males, 43 (11.78%) had drinking history, 58 (15.89%) had a history of surgery, 36 (9.86%) had hypertension, and 6 (1.64%) people with diabetes. Among all participants, 84 subjects were 23.01% of all patients for Grade 0, 142 patients were 38.9% of all patients for Grade 1, 108 patients are 29.59% of all patients for Grade 2, and 31 people were 8.49% of all patients for Grade 3 (Table 1).

**Table 1:** The equilibrium test of training set and testing set

Variable	Total (n = 365)	Group		Statistical magnitude	*P*
		Training set (n = 255)	Testing set (n = 110)		
Age receiving radiotherapy, Mean ± SD	47.69 ± 11.01	47.97 ± 10.83	47.03 ± 11.44	t = − 0.750	0.452
Gender, n (%)				χ^2^ = 0.089	0.765
Female	99 (27.12)	68 (26.67)	31 (28.18)		
Male	266 (72.88)	187 (73.33)	79 (71.82)		
History of drinking, n (%)				χ^2^ = 3.079	0.079
No	322 (88.22)	220 (86.27)	102 (92.73)		
Yes	43 (11.78)	35 (13.73)	8 (7.27)		
History of smoking, n (%)				χ^2^ = 4.120	0.042
No	273 (74.79)	183 (71.76)	90 (81.82)		
Yes	92 (25.21)	72 (28.24)	20 (18.18)		
History of surgery, n (%)				χ^2^ = 0.213	0.644
No	307 (84.11)	213 (83.53)	94 (85.45)		
Yes	58 (15.89)	42 (16.47)	16 (14.55)		
History of hypertension, n (%)				χ^2^ = 0.194	0.660
No	329 (90.14)	231 (90.59)	98 (89.09)		
Yes	36 (9.86)	24 (9.41)	12 (10.91)		
History of diabetes, n (%)				–	1.000
No	359 (98.36)	251 (98.43)	108 (98.18)		
Yes	6 (1.64)	4 (1.57)	2 (1.82)		
T Stage, n (%)				χ^2^ = 1.056	0.788
T1	75 (20.55)	50 (19.61)	25 (22.73)		
T2	72 (19.73)	53 (20.78)	19 (17.27)		
T3	163 (44.66)	115 (45.10)	48 (43.64)		
T4	55 (15.07)	37 (14.51)	18 (16.36)		
N Stage, n (%)				χ^2^ = 2.391	0.495
N0	35 (9.59)	23 (9.02)	12 (10.91)		
N1	147 (40.27)	105 (41.18)	42 (38.18)		
N2	153 (41.92)	103 (40.39)	50 (45.45)		
N3	30 (8.22)	24 (9.41)	6 (5.45)		
Pathological type, n (%)				χ^2^ = 1.056	0.304
A differentiated non-keratinic carcinoma	24 (6.58)	19 (7.45)	5 (4.55)		
An undifferentiated nonkeratinic carcinoma	341 (93.42)	236 (92.55)	105 (95.45)		
Radiotherapy fraction, n (%)				χ^2^ = 0.731	0.393
≤ 30	237 (64.93)	162 (63.53)	75 (68.18)		
> 30	128 (35.07)	93 (36.47)	35 (31.82)		
Dose at 50% of the left parotid volume (Gy), Mean ± SD	25.45 ± 7.26	25.56 ± 7.21	25.21 ± 7.41	t = − 0.41	0.681
Dose at 50% of the right parotid volume (Gy), Mean ± SD	25.81 ± 7.72	26.15 ± 8.11	25.03 ± 6.69	t = − 1.37	0.171
Mean dose to left parotid gland (Gy), Mean ± SD	30.99 ± 5.66	31.13 ± 5.68	30.69 ± 5.65	t = − 0.68	0.495
Mean dose to right parotid gland (Gy), Mean ± SD	31.08 ± 6.08	31.38 ± 6.38	30.39 ± 5.26	t = − 1.53	0.127
Mean dose to oral cavity mean dose (Gy), Mean ± SD	32.65 ± 4.68	32.67 ± 4.71	32.59 ± 4.65	t = − 0.150	0.880
Total radiotherapy dose (Gy), n (%)				χ^2^ = 0.452	0.501
≤ 70GY	277 (75.89)	191 (74.90)	86 (78.18)		
> 70GY	88 (24.11)	64 (25.10)	24 (21.82)		
Mode of radiotherapy-NDP, n (%)				χ^2^ = 0.428	0.513
No	173 (47.40)	118 (46.27)	55 (50.00)		
Yes	192 (52.60)	137 (53.73)	55 (50.00)		
Mode of radiotherapy-DDP, n (%)				χ^2^ = 0.782	0.377
No	286 (78.36)	203 (79.61)	83 (75.45)		
Yes	79 (21.64)	52 (20.39)	27 (24.55)		
Mode of radiotherapy-Others, n (%)				χ^2^ = 0.000	0.989
No	345 (94.52)	241 (94.51)	104 (94.55)		
Yes	20 (5.48)	14 (5.49)	6 (5.45)		
Course of induction chemotherapy, M (Q_1_, Q_3_)	2.00 (2.00, 3.00)	2.00 (2.00, 3.00)	2.00 (2.00, 3.00)	Z = 0.040	0.968
Concomitant chemoradiotherapy, n (%)				χ^2^ = 0.049	0.824
No	77 (21.10)	53 (20.78)	24 (21.82)		
Yes	288 (78.90)	202 (79.22)	86 (78.18)		
Induction chemotherapy, n (%)				χ^2^ = 0.000	0.986
No	30 (8.22)	21 (8.24)	9 (8.18)		
Yes	335 (91.78)	234 (91.76)	101 (91.82)		
The regimens of induction chemotherapy, n (%)				χ^2^ = 2.252	0.895
DP	111 (30.41)	79 (30.98)	32 (29.09)		
DPF	55 (15.07)	35 (13.73)	20 (18.18)		
GP	59 (16.16)	43 (16.86)	16 (14.55)		
None	30 (8.22)	21 (8.24)	9 (8.18)		
Others	4 (1.10)	2 (0.78)	2 (1.82)		
TP	65 (17.81)	47 (18.43)	18 (16.36)		
TPF	41 (11.23)	28 (10.98)	13 (11.82)		
Xerostomia, n (%)				χ^2^ = 1.990	0.574
Grade 0	84 (23.01)	63 (24.71)	21 (19.09)		
Grade 1	142 (38.90)	94 (36.86)	48 (43.64)		
Grade 2	108 (29.59)	76 (29.80)	32 (29.09)		
Grade 3	31 (8.49)	22 (8.63)	9 (8.18)		

*PFS* progression-free survival, *NDP* Nedaplatin, *DDP* cisplatin, *DPF* docetaxel + cisplatin + 5-fluorouracil, *TPF* docetaxel + cisplatin + 5-fluorouracil, *DP* cisplatin + docetaxel, *TP* cisplatin, *GP* gemcitabine + cisplatin, *SD* standard deviation

### The equilibrium test of training set and testing set

As shown in Table 1, no significant difference was observed in age (t = − 0.750, *P* = 0.452), gender (χ^2^ = 0.089, *P* = 0.765), history of drinking (χ^2^ = 3.079, *P* = 0.079), history of surgery (χ^2^ = 0.213, *P* = 0.644), history of hypertension (χ^2^ = 0.194, *P* = 0.660), history of diabetes, T stage (χ^2^ = 1.056, *P* = 0.788), N Stage (χ^2^ = 2.391, *P* = 0.495), pathological type (χ^2^ = 1.056, *P* = 0.304), radiotherapy fraction (χ^2^ = 0.731, *P* = 0.393), dose at 50% of the left parotid gland volume (t = − 0.41, *P* = 0.681), dose at 50% of the right parotid gland volume (t = − 1.37, *P* = 0.171), mean dose to left parotid gland (t = − 0.68, *P* = 0.495), mean dose to right parotid gland (t = − 1.53, *P* = 0.127), total radiotherapy dose (χ^2^ = 0.452, *P* = 0.501), radiotherapy modes, course of induction chemotherapy (Z = 0.040, *P* = 0.968), regimen of induction chemotherapy, mean dose to oral cavity (t = − 0.150, *P* = 0.880) between 255 patients from the training set and 110 patients from the testing set.

### Construction and validation of the RF, DTC and XGB models for xerostomia via LASSO regression

**Fig. 2:**
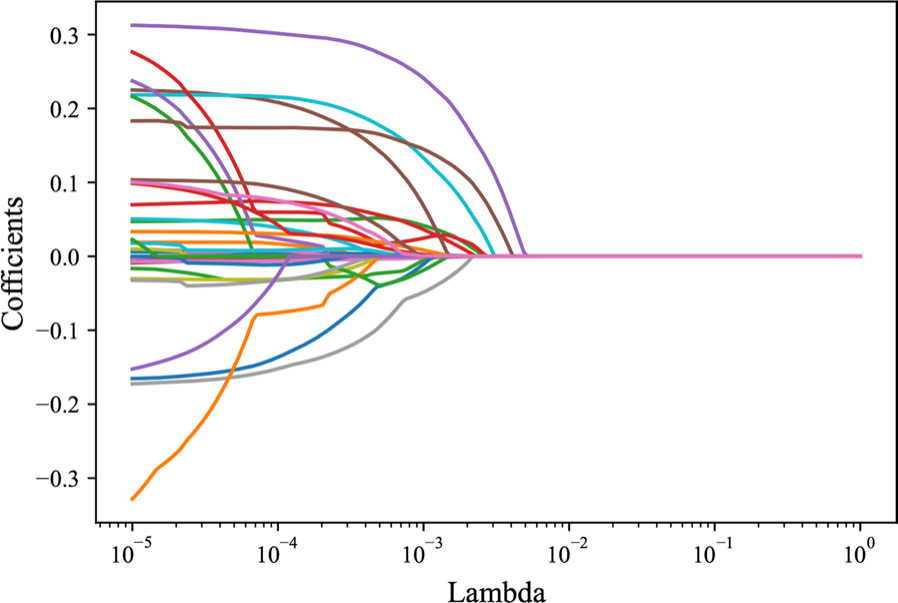
The screen process of predictors for xerostomia via LASSO regression

All the variables were included in the LASSO regression to screen out the predictors for xerostomia in locoregionally advanced NPC patients receiving radical radiotherapy. The results depicted that hypertension, age, total radiotherapy dose, dose at 50% of the left parotid volume, mean dose to right parotid gland, mean dose to oral cavity, and course of induction chemotherapy were potential predictors for xerostomia in locoregionally advanced NPC patients receiving radical radiotherapy (Fig. 2). All the predictors were included for establishing the RF, DTC and XGB models in the training set and verified in the testing set.

### The predictive performance of respective models

**Fig. 3:**
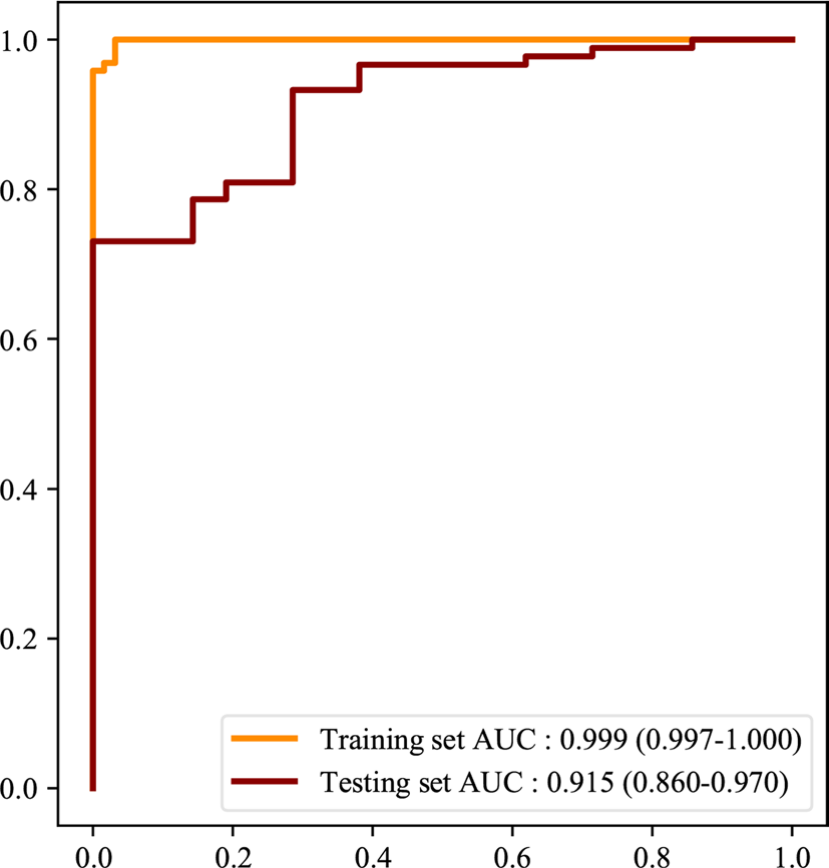
The ROC curve of the RF model for xerostomia

**Fig. 4:**
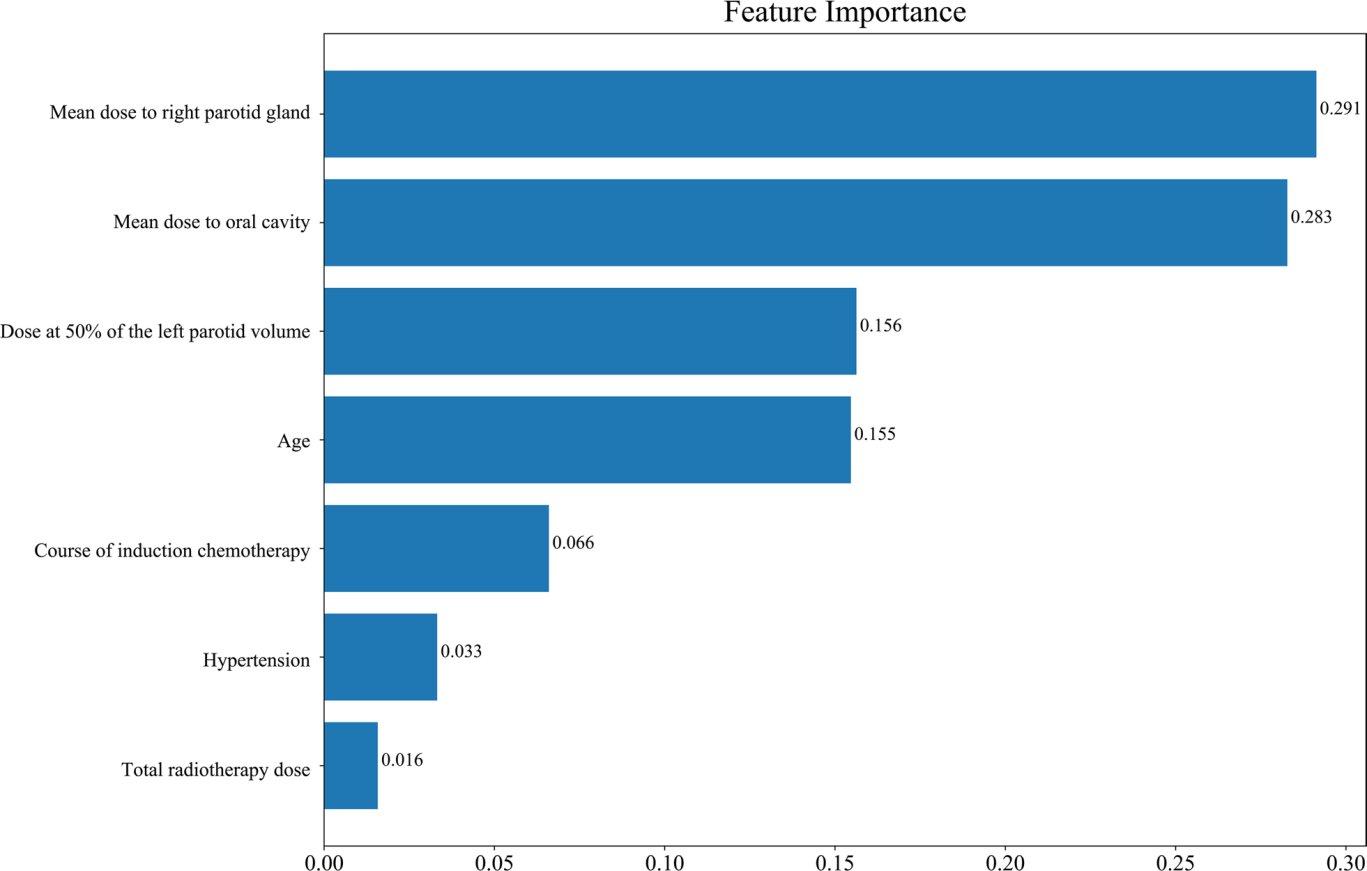
Feature importance diagram of the RF model for xerostomia

As delineated in Table 2, in the RF model, the sensitivity was 1.000 (95%CI 1.000–1.000), the specificity was 0.968 (95%CI 0.925–1.000), the PPV was 0.990 (95%CI 0.975–1.000), the NPV was 1.000 (95%CI 1.000–1.000), the AUC was 0.999 (95%CI 0.997–1.000) and the accuracy was 0.992 (95%CI 0.981–1.000) in the training set. The sensitivity was 0.933 (95%CI 0.880–0.985), the specificity was 0.714 (95%CI 0.521–0.908), the PPV was 0.933 (95%CI 0.880–0.985), the NPV was 0.714 (95%CI 0.521–0.908), the AUC was 0.915 (95%CI 0.860–0.970) and the accuracy was 0.891 (95%CI 0.833–0.949) in the testing set. In DTC model, the sensitivity was 0.943 (95%CI 0.910–0.976), the specificity was 0.984 (95%CI 0.953–1.000), the PPV was 0.995 (95%CI 0.984–1.000), the NPV was 0.849 (95%CI 0.767–0.931), the AUC was 0.963 (95%CI 0.941–0.986), and the accuracy was 0.953 (95%CI 0.927–0.979) in the training set. The sensitivity was 0.775 (95%CI 0.689–0.862), the specificity was 0.762 (95%CI 0.580–0.944), the PPV was 0.932 (95%CI 0.875–0.990), the NPV was 0.444 (95%CI 0.282–0.607), the AUC was 0.769 (95%CI 0.666–0.872), and the accuracy was 0.773 (95%CI 0.694–0.851) in the testing set. In the XGB model, the sensitivity was 0.974 (95%CI 0.951–0.996), the specificity was 0.968 (95%CI 0.925–1.000), the PPV was 0.989 (95%CI 0.975–1.000), the NPV was 0.924 (95%CI 0.860–0.988), the AUC was 0.995 (95%CI: 0.989–1.000) and the accuracy was 0.973 (95%CI 0.952–0.993) in the training set. The sensitivity was 0.820 (95%CI 0.740–0.900), the specificity was 0.714 (95%CI 0.521–0.908), the PPV was 0.924 (95%CI 0.866–0.982), the NPV was 0.484 (95%CI 0.308–0.660), the AUC was 0.834 (95%CI 0.753–0.916) and the accuracy was 0.800 (95%CI 0.725–0.875) in the testing. The sensitivity, NPV in the XGB model and NPV, AUC and accuracy in the DTC model were statistically lower than the RF model in the training set. The sensitivity, PPV, NPV, AUC and accuracy in the DTC model was lower than in the RF model in the testing set, so the RF model was finally selected as the prediction model in this study. The ROC curve of the RF model was shown in Fig. 3. Feature importance diagram revealed that mean dose to right parotid gland, mean dose to oral cavity and dose at 50% of the left parotid volume were important variables associated with the occurrence of xerostomia in locoregionally advanced NPC patients receiving radical radiotherapy (Fig. 4).

**Table 2:** The predictive values of the models

	Sensitivity (95%CI)	Specificity (95%CI)	PPV (95%CI)	NPV (95%CI)	AUC (95%CI)	Accuracy (95%CI)
*Training set*						
RF	1.000 (1.000–1.000)	0.968 (0.925–1.000)	0.990 (0.975–1.000)	1.000 (1.000–1.000)	0.999 (0.997–1.000)	0.992 (0.981–1.000)
XGB	0.974 (0.951–0.996)*	0.968 (0.925–1.000)	0.989 (0.975–1.000)	0.924 (0.860–0.988)*	0.995 (0.989–1.000)	0.973 (0.952–0.993)
DTC	0.943 (0.910–0.976)	0.984 (0.953–1.000)	0.995 (0.984–1.000)	0.849 (0.767–0.931)*	0.963 (0.941–0.986)*	0.953 (0.927–0.979)*
*Testing set*						
RF	0.933 (0.880–0.985)	0.714 (0.521–0.908)	0.933 (0.880–0.985)	0.714 (0.521–0.908)	0.915 (0.860–0.970)	0.891 (0.833–0.949)
XGB	0.820 (0.740–0.900)	0.714 (0.521–0.908)	0.924 (0.866–0.982)	0.484 (0.308–0.660)	0.834 (0.753–0.916)	0.800 (0.725–0.875)
DTC	0.775 (0.689–0.862)^#^	0.762 (0.580–0.944)	0.932 (0.875–0.990)^#^	0.444 (0.282–0.607)^#^	0.769 (0.666–0.872)^#^	0.773 (0.694–0.851)^#^

*RF* random forest, *DTC* decision tree classifier, *XGB* extreme-gradient boosting, *PPV* positive predictive value, *NPV* negative predictive value, *AUC* area under the curve

^*^Compared with the training set in the RF model, the difference was statistically different

^#^Compared with the testing set in the RF model, the difference was statistically different

### Prediction of patients with grade 3 xerostomia in those with high risk of xerostomia

**Fig. 5:**
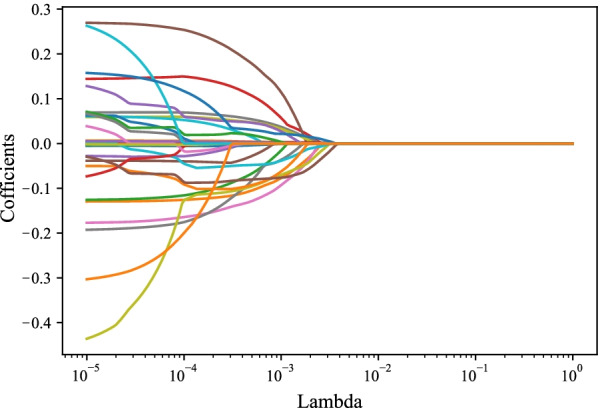
The screen process of predictors for xerostomia Grade 3 via LASSO regression

**Fig. 6:**
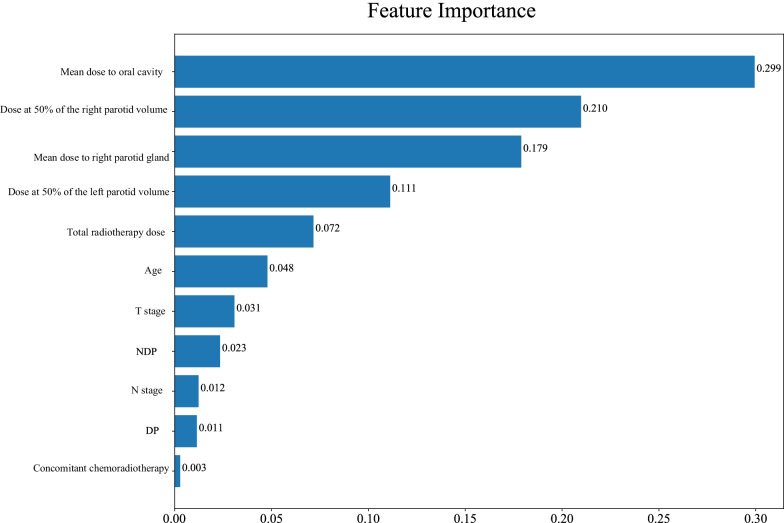
Feature importance diagram of the RF model for xerostomia Grade 3

**Fig. 7:**
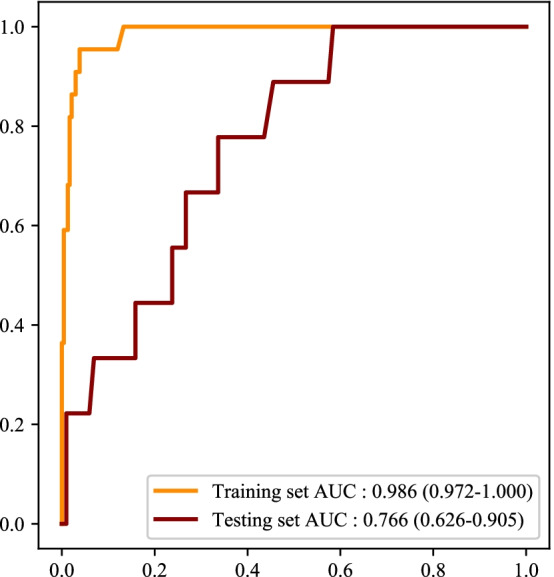
The ROC curve of the RF model for xerostomia Grade 3

As exhibited in Fig. 5, the predictors for patients with Grade 3 xerostomia were screened by LASSO regression. The predictors included age, T stage, N stage, dose at 50% of the left parotid volume, dose at 50% of the right parotid volume, mean dose to right parotid gland, mean dose to oral cavity, concomitant chemoradiotherapy or not, NDP, DP, and total radiotherapy dose (Fig. 6). The prediction model for Grade 3 xerostomia in those with high risk of xerostomia was established based on these predictors. The results delineated that the RF model showed the best predictive ability with the AUC of 0.986 (95%CI 0.972–1.000) in the training set, and 0.766 (95%CI 0.626–0.905) in the testing set (Fig. 7, Table 3).

**Table 3:** The predictive performance of models for Grade 3 xerostomia

	Sensitivity (95%CI)	Specificity (95%CI)	PPV (95%CI)	NPV (95%CI)	AUC (95%CI)	Accuracy (95%CI)
*Training set*						
RF	0.955 (0.868–1.000)	0.961 (0.937–0.986)	0.700 (0.536–0.864)	0.996 (0.987–1.000)	0.986 (0.972–1.000)	0.961 (0.937–0.985)
XGB	0.864 (0.720–1.000)	0.858 (0.814–0.903)	0.365 (0.235–0.496)	0.985 (0.969–1.000)	0.914 (0.844–0.984)	0.859 (0.816–0.902)
DTC	0.500 (0.291–0.709)	0.991 (0.980–1.000)	0.846 (0.650–1.000)	0.955 (0.928–0.981)	0.746 (0.639–0.853)	0.949 (0.922–0.976)
*Testing set*						
RF	0.333 (0.025–0.641)	0.851 (0.782–0.921)	0.167 (0.000–0.339)	0.935 (0.884–0.985)	0.766 (0.626–0.905)	0.809 (0.736–0.883)
XGB	0.444 (0.120–0.769)	0.792 (0.713–0.871)	0.160 (0.016–0.304)	0.941 (0.891–0.991)	0.661 (0.478–0.843)	0.764 (0.684–0.843)
DTC	0.222 (0.000–0.494)	0.980 (0.953–1.000)	0.500 (0.010–0.990)	0.934 (0.887–0.981)	0.601 (0.457–0.746)	0.918 (0.867–0.969)

*RF* random forest, *DTC* decision tree classifier, *XGB* extreme-gradient boosting, *PPV* positive predictive value, *NPV* negative predictive value, *AUC* area under the curve

## Discussion

In this study, the clinical data of locoregionally advanced NPC 365 patients who underwent radical radiotherapy were collected to analyze the predictive factors of xerostomia, and established prediction models for xerostomia and Grade 3 xerostomia in locoregionally advanced NPC patients who underwent radical radiotherapy. The findings delineated that hypertension, age, total radiotherapy dose, dose at 50% of the left parotid volume, mean dose to right parotid gland, mean dose to oral cavity, and course of induction chemotherapy were associated with the risk of xerostomia. The prediction models for xerostomia all had a good predictive ability for distinguishing xerostomia patients from non-xerostomia patients and the RF model showed the best predictive performance. The RF model presented good predictive value in predicting patients who at high risk of Grade 3 xerostomia in those with high risk of xerostomia.

Radiotherapy for NPC is challenging due to the proximity of the post-nasal space to many critical organs such as salivary glands, and the damage of radiation to these salivary glands often results in long-term morbidity [25]. Xerostomia is a complication due to the radiotherapy damage to the salivary glands in NPC patients, which seriously influence the quality of lives in those patients [26]. Radiotherapies in NPC patients can affect the secretion of the salivary glands and the radiation dose > 40 Gy can cause irreversible loss of salivary gland function [27]. Previous studies also found that radiotherapy may change the protein levels as well as the concentration of electrolytes in saliva including sodium and chloride [28, 29]. To quickly identify patients with high risk of xerostomia in NPC patients receiving radiotherapies is of great value in clinic. In the current study, hypertension was a potential predictor for xerostomia in NPC patients receiving radiotherapies. Hypertension is reported to be correlated with the poor overall survival outcome in NPC patients [30]. Hypertension may result in arteriosclerosis, and sclerosis and stenosis of the arterioles may cause the degeneration and hypofunction of some organs including parotid gland and oral cavity [31]. The salivary flow rate and its pH were influenced by hypertension and the salivary flow rate was lower in borderline hypertension people than in normotensives [32, 33]. According to previous study, drugs utilized for controlling hypertension also have a potential to induce xerostomia [34, 35]. Drugs controlling hypertension act on central alpha 2 adrenergic receptors, and the activation of alpha 2 adrenoceptor is in the lateral hypothalamus which is an important central area for the control of salivary secretion and resulting in xerostomia [36]. Herein, the total radiotherapy dose was associated with the occurrence of xerostomia in NPC patients after radical radiotherapy. With the increase of the frequency and dose of radiotherapy, radiotherapy will have more influence on the physiological structure and function of other organs in the head and neck of patients with NPC, and the number and severity of complications will increase [37]. One of the effective ways to reduce the injury of parotid gland function is to reduce the volume or dose to parotid gland exposure [38]. Previously, several studies also demonstrated that for patients with residual tumor after conventional external irradiation dose of 70–72 Gy, the same efficacy can be achieved in patients; the irradiation dose to the surrounding normal tissues as well as the occurrence of radiotherapy sequelae can be reduced using after-load radiotherapy or 3-dimensional conformal radiation therapy to ≥ 80 Gy [38, 39]. The increased radiation dose to oral cavity was associated with an elevated risk of xerostomia in head and neck squamous cell carcinoma after curative intended radiotherapy [40]. These conclusions supported the finding in our study. We identified that the increased mean radiation dose to oral cavity was associated with the risk of xerostomia in locoregionally advanced NPC patients receiving radical radiotherapy. The mean dose to the parotid gland was the most important factor that influenced the parotid function [41]. Teshima et al. found that the parotid gland function might be impaired and the salivary secretion was significantly decreased when the parotid gland received a total of 30 Gy irradiation, when the parotid gland received more than 40 Gy irradiation, the parotid gland stopped secreting saliva, and when the parotid gland received more than 75 Gy irradiation, the acinar cells of parotid gland would be necrotic [42]. These evidence supported the findings in our study, which identified that the mean dose to parotid gland was a vital predictor for xerostomia in locoregionally advanced NPC patients receiving radical radiotherapy. Additionally, we found the dose at 50% of the left parotid volume was also associated with the occurrence of xerostomia in locoregionally advanced NPC patients receiving radical radiotherapy. Previously, clinicians found that the volume of parotid gland differs in different patients, and those with larger volume of parotid gland might have more acinous cells and may have better protection against radiation damage than those with small parotid gland [43]. Therefore, a former study indicated that a larger volume of the parotid gland was a protective factor for xerostomia [44]. Induction chemotherapy is widely applied for NPC patients in China, and multiple studies have uncovered that induction chemotherapy might be associated with increased risk of xerostomia in NPC patients. Liu et al. conducted a study compared the efficiency and safety of induction chemotherapy plus concomitant chemoradiotherapy versus induction chemotherapy plus volumetric modulated arc therapy alone in the treatment of stage II-IVB NPC patients, and found that 34.53% or 48.72% patients had xerostomia, respectively [45]. This was allied with the data in the current study, showing that the course of induction chemotherapy was an important predictor of xerostomia in NPC patients. Other studies also indicated that chemotherapy was not associated with xerostomia [46]. This difference may be because the inclusion and exclusion criterion of studies was not the same. Older age was a risk factor for xerostomia in many patients [47, 48]. This provide evidence to the results in our study, which identified that the risk of age was associated with the occurrence of xerostomia in locoregionally advanced NPC patients receiving radical radiotherapy.

Herein, several prediction models for xerostomia in locoregionally advanced NPC patients receiving radical radiotherapy were established in the training set and the validation of the models were conducted in the testing set. To our knowledge, it is the first prediction model for xerostomia in locoregionally advanced NPC patients receiving radical radiotherapy. The predictive values of the models were evaluated and all models present good predictive performance for xerostomia in locoregionally advanced NPC patients receiving radical radiotherapy. In addition, the predictive values were compared between the models, and the RF model was best model with an AUC of 0.999 in the training set and 0.995 in the testing set. The sensitivity, specificity, PPV, NPV and accuracy of the model were all good. The RF model was selected as the final model. These data indicated that this prediction model had a good predictive value, which might be worthy for predicting xerostomia in locoregionally advanced NPC patients receiving radical radiotherapy in clinic and providing timely prevention in those patients. In those who were predicted to have high risk of xerostomia, we constructed several models for predicting xerostomia Grade 3. RF also showed good predictive ability. This might provide a tool for identifying patients who with a high risk of severe xerostomia, and provide appropriate treatments in those patients to prevent severe xerostomia and timely interventions should be applied to improve their prognosis.

This study had several limitations. Firstly, the prediction models were established based on data obtained from a single center and required external validation in another cohort. Secondly, the dosimetric parameters of parotid saliva flow rate of patients were not measured, and the evaluation of xerostomia in patients could not be quantified, which may cause selection bias in our study. This study assessed xerostomia according to the RTOG/EORTC system. The subjective assessment of the RTOG/EORTC system may underestimate the severity of xerostomia [49, 50]. Thirdly, patient-reported toxicities may be different from physician-reported toxicities. The treating physician assigns the grade depending on the toxicities, which might result in the inter-observer differences. Fourthly, the detailed medication history of patients was not recorded in patients, and whether the medical history had influence on the occurrence of xerostomia in locoregionally advanced NPC patients receiving radical radiotherapy remains to be explored in the future. The findings of this study should be verified in a large scale of prospective study with more reliable evaluation of xerostomia.

## Conclusions

This study collected the clinical data of 365 patients with locoregionally advanced NPC who underwent radical radiotherapy. The predictors of xerostomia in patients with locoregionally advanced NPC who underwent radical radiotherapy were analyzed and identified that a history of hypertension, age, total radiotherapy dose, dose at 50% of the left parotid volume, mean dose to right parotid gland, mean dose to oral cavity, and course of induction chemotherapy. The RF model for predicting xerostomia was established based on the predictors and had good predictive ability. The RF model for predicting the risk of severe xerostomia also showed good predictive performance. The findings of the current study might provide a reference for identify patients with high risk of xerostomia and severe xerostomia in locoregionally advanced NPC who underwent radical radiotherapy and provide early interventions to reduce the occurrence of xerostomia or severe xerostomia.

## Data Availability

The datasets used and/or analyzed during the current study are available from the corresponding author on reasonable request.

## Abbreviations

NPC: Nasopharyngeal carcinoma
HR: Hazars ratio
IMRT: Intensity modulated radiation therapy
EORTC: The European Organization for Research and Treatment of Cancer
PFS: Progression-free survival
NDP: Nedaplatin
DDP: Cisplatin
DPF: Docetaxel + cisplatin + 5-fluorouracil
TPF: Docetaxel + cisplatin + 5-fluorouracil
DP: Cisplatin + docetaxel
TP: Cisplatin
GP: Gemcitabine + cisplatin
RF: Random forest
DTC: Decision tree classifier
XGB: Extreme-gradient boosting
PPV: Positive predictive value
NPV: Negative predictive value
AUC: Area under the curve
SD: Standard deviation
